# *In utero* exposure to persistent organochlorine pollutants and reproductive health in the human male

**DOI:** 10.1530/REP-13-0488

**Published:** 2014-12

**Authors:** Anne Vested, Cecilia H Ramlau-Hansen, Sjurdur F Olsen, Jens Peter Bonde, Henrik Støvring, Susanne L Kristensen, Thorhallur I Halldorsson, Panu Rantakokko, Hannu Kiviranta, Emil H Ernst, Gunnar Toft

**Affiliations:** 1 Department of Occupational Medicine, Danish Ramazzini Centre, Aarhus University Hospital, Noerrebrogade 44 Building 2C, DK-8000 Aarhus C, Denmark; 2 Section for Epidemiology, Department of Public Health, University of Aarhus, Bartholins Allé 2 Building 1260, DK-8000 Aarhus C, Denmark; 3 Statens Serum Institut, Centre for Fetal Programming Artellerivej 5, DK-2300 Copenhagen S, Denmark; 4 Department of Occupational and Environmental Medicine, Bispebjerg Hospital of Copenhagen University, Bispebjerg Bakke 23 Building 33 1st floor, DK-2400 Copenhagen NV, Denmark; 5 Department of Public Health, Biostatistics, Bartholins Allé 2 Building 1261, DK-8000 Aarhus C, Denmark; 6 Faculty of Food Science and Nutrition, University of Iceland, Eiríksgata 29, 101 Reykjavík, Iceland; 7 Department of Environmental Health, National Institute for Health and Welfare (THL), PO Box 95, FI-70701 Kuopio, Finland; 8 Section West, Institute for Biomedicine, Aarhus University, Ole Worms Allé 2 building 1170, 8000 Aarhus C, Denmark

## Abstract

Persistent organochlorine pollutants (POPs) are ubiquitous, bioaccumulative compounds with potential endocrine-disrupting effects. They cross the placental barrier thereby resulting in *in utero* exposure of the developing fetus. The objective of this study was to investigate whether maternal serum concentrations of polychlorinated biphenyls (PCBs) and *p*,*p*′-dichlorodiphenyldichloroethylene (*p*,*p*′-DDE) during pregnancy are associated with son's semen quality and reproductive hormone levels. During 2008–2009, we recruited 176 male offspring from a Danish cohort of pregnant women who participated in a study in 1988–1989. Each provided semen and blood samples that were analyzed for sperm concentration, total sperm count, motility, and morphology, and reproductive hormone levels, respectively. The maternal blood samples were collected in pregnancy week 30 and were analyzed for the concentrations of six PCBs (PCB-118, -138, -153, -156, -170, and -180) and *p*,*p*′-DDE. The potential associations between *in utero* exposure to ΣPCBs (pmol/ml), Σdioxin like-(DL) PCBs (PCB-118 and -156) (pmol/ml), and *p*,*p*′-DDE and semen quality and reproductive hormone levels were investigated using multiple regression. Maternal median (range) exposure levels of ΣPCB, ΣDL-PCB, and *p*,*p*′-DDE were 10.0 (2.1–35.0) pmol/ml, 0.8 (0.2–2.7) pmol/ml, and 8.0 (0.7–55.3) pmol/ml, respectively, reflecting typical background exposure levels in the late 1980s in Denmark. Results suggested that *in utero *exposure to ΣPCB, ΣDL-PCB, and *p*,*p*′-DDE was not statistically significantly associated with semen quality measures or reproductive hormone levels. Thus, results based on maternal PCB and *p*,*p*′-DDE concentrations alone are not indicative of long-term consequences for male reproductive health; however, we cannot exclude that these POPs in concert with other endocrine-modulating compounds may have adverse effects.

## Introduction

Persistent organochlorine pollutants (POPs) such as polychlorinated biphenyls (PCBs) and organochlorine pesticides such as dichlorodiphenyltrichloroethane (DDT) are ubiquitous, organic pollutants that have been widely used until they were banned in Western countries in the late 1970s. Because of their lipophilic structure, they accumulate in adipose tissue and bioaccumulate in the food chain (Safe 1990, Cook *et al*. 2011) and, therefore, humans are at present mainly exposed to these POPs through diet, for example through consumption of fatty fish, dairy products, and meat (Gasull *et al*. 2011). The compounds are persistent and have long half-lives in human tissue (Wolff *et al*. 2000, Seegal *et al*. 2011), and can be detected in humans worldwide due to their wide distribution and usage. Many PCB congeners have been shown to display hormone-modulating activities, and the DDT-degradation product *p*,*p*′-dichlorodiphenyldichloroethylene (*p*,*p*′-DDE) is regarded a potent androgen receptor antagonist (Kelce *et al*. 1995). Both groups of substances readily cross the placental barrier (Saxena *et al*. 1981, Jacobson *et al*. 1984), resulting in *in utero* exposure of the developing fetus. In addition, substantial postnatal exposure results from breastfeeding, whereby accumulated compounds are transferred from the mother to the infant.

Low sperm concentration is associated with a longer time to pregnancy and a sperm concentration of 40×10^6^/ml is considered a threshold below which fecundity declines (Bonde *et al*. 1998). A recent study from an ongoing surveillance of semen quality in young Danish men reporting for military draft (1996–2010) has suggested that only around 23% of young Danish men have an optimal sperm concentration of above 40×10^6^/ml and more than 9% morphologically normal spermatozoa (Jørgensen *et al*. 2012). Furthermore, studies suggest that young Danish men, alongside young men from Norway and Germany, may have lower sperm concentrations than young men in other European countries (Jørgensen *et al*. 2002, Punab *et al*. 2002, Richthoff *et al*. 2002, Paasch *et al*. 2008, Axelsson *et al*. 2011, Fernandez *et al*. 2011). Together with indications of increasing incidences of testicular cancer and congenital malformations such as cryptorchidism and hypospadias, this has led to the hypothesis that exposure to endocrine-modulating compounds during fetal life could cause an imbalance in the fetal hormonal environment and affect normal development of male reproductive organs, thereby causing long-term effects on male reproductive health (Toppari *et al*. 1996, Toppari & Skakkebaek 1998, Skakkebaek *et al*. 2001, Sharpe *et al*. 2003).

Cross-sectional studies have indicated an inverse association between serum levels of PCB153, PCB138, and *p*,*p*′-DDE exposure in adult life and sperm motility (Rozati *et al*. 2002, Hauser *et al*. 2003, Richthoff *et al*. 2003, Toft *et al*. 2006), and studies of men living in areas sprayed with DDT have suggested inverse associations between *p*,*p*′-DDE and sperm motility, semen volume, total sperm count, and positive associations with sperm tail defects and poor sperm chromatin condensation (De Jager *et al*. 2006, Aneck-Hahn *et al*. 2007).

In addition, the Yu Cheng oil-disease provided insights into the consequences of *in utero* exposure to high levels of PCBs. These included increased abnormal sperm morphology, decreased sperm motility, and reduced oocyte penetration capacity among *in utero* exposed cases (Guo *et al*. 2000).

Based on the hypotheses that fetal life exposures may affect male reproductive organ development and cause long-term effects on reproductive health, the aim of this study was to conduct a population-based follow-up study to investigate the potential effects of *in utero* exposure to background levels of PCBs and *p*,*p*′-DDE on semen quality and reproductive hormone levels among young postpubertal Danish men born in the years 1988–1989. To our knowledge, this is the first population-based follow-up study to address the associations between *in utero* exposure to PCBs and *p*,*p*′-DDE and semen quality and reproductive hormone levels in early adult life.

## Materials and methods

### Population

A Danish population-based pregnancy cohort was recruited in 1988–1989, when the pregnant women attended a routinely scheduled consultation with a midwife in Aarhus, Denmark, at pregnancy week 30. They answered a detailed questionnaire, were interviewed on dietary and lifestyle habits, and provided a blood sample for storage in a biobank. Of all 1212 pregnant women invited, 965 (80%) participated (Olsen *et al*. 1995*a*, 1995*b*).

**Figure 1:**
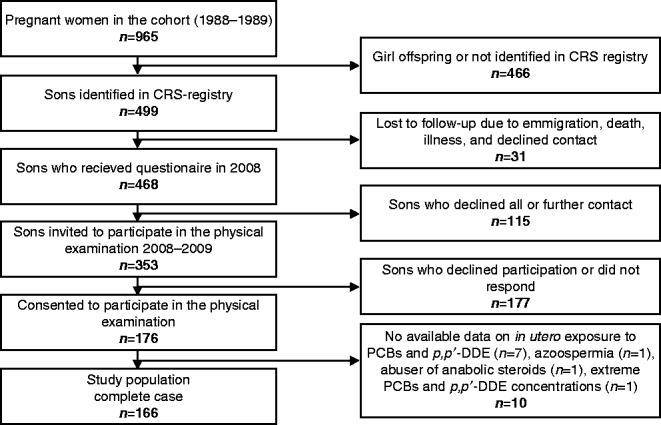
Flow chart for recruitment of participants.

The follow-up for the male offspring of this cohort was initiated in 2008, where the sons were invited to fill in an internet-based questionnaire on health and lifestyle habits. In 2008–2009, the sons were invited to participate in a physical examination at Aarhus University Hospital, Denmark, where they provided a semen sample, a blood sample, and self-estimated testicular volume using an orchidometer. One hundred and seventy-six sons attended the physical examination, corresponding to a participation rate of 38% of all male offspring in the source cohort (Fig. 1). All participants signed a written informed consent before participation, and the study was approved by the regional ethics committee (registration number M-20010157).

Two participants were excluded from all statistical analyses due to azoospermia and abuse of anabolic steroids respectively. In addition, one participant was excluded due to an extremely high maternal serum level of PCBs and *p*,*p*′-DDE (PCB-153 concentration: 12 ng/ml and *p*,*p*′-DDE concentration: 32 ng/ml) compared with the remaining participants, leaving a study population of 173 available for this study.

### Data collection

The participants attended the physical examinations from February 2008 until September 2009. The young men were instructed to collect semen samples into plastic containers by masturbation, at their residence or at the hospital, and to keep the samples close to the body during transport. At the hospital, semen samples were stored in a heating chamber (37 °C) until semen analysis. All participants reported time and date of the semen sample collection and information on potential spillage during semen sample collection. The participants were instructed to self-measure their testicular volumes by using a Prader orchidometer and finding the volume on the orchidometer, that best corresponded to the size of the testicle, going from the smallest towards the largest volume on the orchidometer. This method has previously been reported to be a valid method for measurement of testicular size (Ramlau-Hansen *et al*. 2007b). In addition, height and weight were measured for each participant by an examiner.

The blood samples were taken between 0730 and 1330 h. The participants were instructed to present at the physical examination being in a fasted state for 10 h.

### Semen analysis

The semen analysis was initiated within 1 h from ejaculation for 86% of the participants and all semen samples were analyzed within 2 h from ejaculation. Conventional semen analysis was performed according to World Health Organization (WHO) guidelines from 1999 (World Health Organization 1999) and the Nordic Association for Andrology Manual on Basic Semen Analysis. The sperm concentration was assessed on two dilutions of the semen sample using a Neubauer counting chamber and the final sperm concentration calculated from the sum of the two counts divided by a factor derived from the dilution factor and the number of Neubauer counting chamber squares counted. Sperm motility was assessed in an aliquot of 6 μl semen by classifying spermatozoa into four motility categories by counting the proportion of rapidly progressive (>25 μm/s), slowly progressive (5–25 μm/s), non-progressive (<5 μm/s), and immotile spermatozoa for 2×100 spermatozoa. This was repeated in a new aliquot of 6 μl semen and, hence, a total of 400 spermatozoa were classified per semen sample. Additional information is shown in the study reported by Vested *et al*. (2011). Furthermore, sperm concentration and motility were assessed by computer-assisted semen analysis (CASA), using the Copenhagen Rigshospitalet Image House Sperm Motility Analysis System (CRISMAS) Clinical software version 4.6 (Image House Medical, IHMedical A/S, Copenhagen, Denmark), as previously described (Vested *et al*. 2011). For both conventional and CASA motility assessment, participants with missing information on motility assessments were assigned a mean of each of the motility categories based on the participants with information on sperm motility. The laboratory attended the Special Interest Group in Andrology ESHRE Subcommittee External Quality Assurance control program, and all tests were within the central 50% of the reference results. The sperm morphology was assessed using ‘Strict criteria’ (Menkveld *et al*. 1990). The assessments of morphology were only included in the statistical analyses for the participants where quality control status of the sperm morphology smears were in agreement with the ‘strict criteria’.

### Blood sample analyses

The maternal serum samples were collected in pregnancy week 30 in 1988–1989 and stored at −20 °C until analysis in 2011. The concentrations of *p*,*p*′-DDE and six prevalent PCB congeners (PCB-118, -138, -153, -156, -170, and -180) were measured from 200 μl aliquots of maternal serum by liquid–liquid extraction followed by gas chromatography coupled with high resolution mass spectometry at the National Institute for Health and Welfare (THL), Department of Environmental Health, Kuopio, in Finland according to a sample pretreatment method described by Koponen *et al*. (2013). Two blanks and two control samples (NIST SRM 1589a) were included in each batch of samples (*n*=25) in order to control for laboratory contamination and accuracy, and precision of the method respectively. The average recoveries of measured POPs in the control samples were 97–106% of the certified values. The between-assay coefficient of variation (CV) was 4.0% (at 0.17 ng/ml) for PCB-118; 4.2% (at 0.54 ng/ml) for PCB-138; 2.7% (at 0.97 ng/ml) for PCB-153; 6.5% (at 0.08 ng/ml) for PCB-156; 6.7% (at 0.21 ng/ml) for PCB-170; 2.6% (at 0.53 ng/ml) for PCB-180; and 2.1% (at 11.2 ng/ml) for *p*,*p*′-DDE. The limits of quantification (LOQs) were between 2 and 5 pg/ml. The concentrations of the six PCB congeners and *p*,*p*′-DDE were above LOQ in all samples.

In offspring blood samples, serum concentrations of LH, FSH, estradiol, and testosterone were analyzed using immunoassays (cobas 6000 e601, Roche Diagnostics) at the Department of Clinical Biochemistry, Aarhus University Hospital, Denmark with CVs being 1.1–2.4, 1.9–2.1, 1.5–2.9, and 2.2–4.5%, respectively, and concentrations of sex hormone-binding globulin (SHBG) were measured using a solid-phase two-site chemiluminescent immunometric assay (IMMULITE 2000, Siemens Healthcare Diagnostics Products Ltd., Gwynedd, UK) with a CV of 4.5–4.7%. Inhibin-B was measured undiluted using a commercially available ELISA (Oxford Bio-innovation Ltd, Oxfordshire, England, UK) with a detection of 20 pg/ml and a CV below 7% at the Laboratory of Reproductive Biology, Juliane Marie Centre for Women, Children and Reproduction, University Hospital of Copenhagen, Denmark. The measurements below the detection limit for estradiol (*n*=3) were recoded to half the detection limit (0.025 nmol/l).

### Statistical analysis

We converted each of the measured PCB congeners into molar concentrations by dividing PCBs in pg/ml by the congener's molecular weight (g/mol), and PCB congeners were summed in two exposure groupings: i) Σ all measured PCBs (pmol/ml) (ΣPCBs), and ii) Σ dioxin-like PCBs (PCB-118 and PCB-156) (ΣDL-PCBs) (pmol/ml). In addition, *p*,*p*′-DDE concentration in maternal blood samples was selected as a biomarker of organochlorine pesticide exposure and converted into molar concentration (pmol/ml). Each exposure variable was divided into three exposure groups (low, medium, and high) based on exposure tertiles. The outcome variables included semen parameters (sperm concentration, total sperm count, semen volume, percentage of progressive spermatozoa (rapidly progressive and slowly progressive), percentage of motile spermatozoa (rapidly progressive, slowly progressive, and non-progressive spermatozoa), and percentage of morphologically normal spermatozoa, mean right and left testicular volume, and reproductive hormones (testosterone, free testosterone calculated as suggested by Vermeulen *et al*. (1999), estradiol, LH, FSH, SHBG, and inhibin B).

The crude differences between tertile exposure groups and linear trends on continuous ΣPCBs, ΣDL-PCBs, and *p*,*p*′-DDE concentrations and the studied outcomes (untransformed) were assessed by linear regression analysis with robust variance estimates. The adjusted trends were tested by multiple regression analysis, by entering the summed PCB levels or *p*,*p*′-DDE levels as continuous variables.

The outcome variables were natural logarithm (ln) transformed before multiple regression analysis, and low PCB groups or low *p*,*p*′-DDE group were used as referents. The results from the multiple regression analyses are presented as relative differences on the original measurement scale with 95% confidence intervals (95% CI). All multiple regression results were adjusted for the history of reproductive tract disease (inguinal hernia, varicocele, testicular hydrocele, incarcerated hernia, phimosis, torsio testis, clamydia, gonorrhea, and epididymitis combined into a combined into one variable, yes/ no), sons' BMI, (kg/m^2^) (Sermondade *et al*. 2013), sons' smoking status (current and occasional smoker/ ex and never smoker) (Ramlau-Hansen *et al*. 2007*c*), maternal smoking during pregnancy (yes/ no) (Ramlau-Hansen *et al*. 2007*a*), socioeconomic status (total annual income for the household in 1987 (<200 000 DKK/≥200 000 DKK)), and maternal serum total lipid concentration (continuously in g/l). Total lipid concentration in maternal serum (g/l) was calculated using the following equation for estimation of total lipid based on the information on serum cholesterol and triglycerides: total lipid=0.96+1.28×(cholesterol (g/l)+triglycerides (g/l)). For this calculation, the average molecular weights of cholesterol and triglycerides were assumed to be 571 and 807 g/mol, respectively, based on the assumption that the proportion of free and esterified cholesterol was 1:2 (Rylander *et al*. 2006). In addition, results on sperm concentration, total sperm count, percentage progressive spermatozoa, percentage motile spermatozoa, semen volume, and testicular volume were adjusted for abstinence time (≤48 h/≥49 h); sperm concentration was adjusted for spillage during semen sample collection (yes/ no); percentage of progressive spermatozoa and percentage of motile spermatozoa were adjusted for time from ejaculation to semen analysis (continuous, minutes); and the reproductive hormones were adjusted for time of the day of blood sampling (0730–0929 h/0930–1129 h/≥1130 h). The participants who reported spillage during semen sample collection were excluded from multiple regression analysis on total sperm count and semen volume. All statistical analyses were performed using Stata 12.1 software (Stata Corporation, College Station, TX, USA).

### Multiple imputation analyses

Multiple imputation was used to handle missing data under the assumption that data fulfilled the missing at random criteria (Sterne *et al*. 2009).

The data on the exposure variables were missing on seven participants due to missing maternal blood samples or inadequate blood sample volume for POP concentration measurements. The numbers of participants with missing data in the outcome variables ranged between two and eight and the number of participants with missing values in the co-variates ranged between 0 and 15.

For the main imputation model, 100 datasets were imputed with values for missing data. Before imputation, assumptions of normal distributions of the residuals of all continuous variables imputed on the linear scale in the multiple imputation model were checked and confirmed. We used the best transformations of the outcome variables for the multiple imputation of missing values (sperm concentration: cubic root; total sperm count, semen volume, and inhibin B: square root; progressive motility and all motility: squared; FSH: ln; and morphologically normal spermatozoa, LH, estradiol, testosterone, free testosterone, and SHBG; no transformation). Imputations for the main model were based on including all outcomes, exposure variables, and potential confounders mentioned above plus fever within the last three months (participants), maternal pre-pregnancy BMI, parity, maternal alcohol consumption-, maternal educational level, and maternal serum hexachlorbenzene level during pregnancy in the predictive models. In addition, interval censoring was allowed for variables that were imputed on a continuous scale according to plausible ranges predicted from the ranges within the dataset and limits of detection. All imputations and corresponding analyses were performed using Stata 12.1.

Sensitivity analyses with different imputation models were performed to check for consistency in results under different imputation models, but no discernible differences were observed in results (details available upon request).

## Results

The 173 male offspring participating in the follow-up study had a median (range) age of 20 (19–21) years. Complete case data on maternal pregnancy week 30 serum concentrations (ng/ml) and lipid-adjusted serum concentrations (ng/g lipid) of *p*,*p*′-DDE, and the measured PCB congeners (PCB-118, -138, -153, -156, -170, and -180) are given in Table 1, and characteristics of the participants according to tertiles of ΣPCB and *p*,*p*′-DDE exposure are shown in Table 2. Maternal serum lipid concentrations and sons' abstinence time differed substantially among the exposure groups. The mothers in the lowest ΣPCB tertile had statistically significantly lower serum lipid levels than mothers in the highest tertile, and more participants in the lowest ΣPCB- and *p*,*p*′-DDE tertiles had been sexually abstinent for more than 2 days compared with the high ΣPCB tertile and both medium and high *p*,*p*′-DDE tertiles (Table 2).

The results from the main multiple imputation model showed no consistent associations between any of the exposure variables (ΣPCB, ΣDL-PCB, or *p*,*p*′-DDE) and the studied outcomes for either crude or adjusted data (Tables 3, 4, and 5). However, in crude models, there were statistically significantly lower testosterone, free testosterone, estradiol, and LH levels in the medium ΣPCB exposure group compared with the reference group (low), which were corroborated by the adjusted analyses, although not statistically significant for LH (Table 3). In addition, sons in the medium *p*,*p*′-DDE exposure tertile had significantly lower semen volume and percentage of progressive spermatozoa compared with the reference group, but differences between the reference group and the sons exposed to the highest *p*,*p*′-DDE tertile were not statistically significant. After adjustment, differences between the reference groups and the medium exposure groups attenuated and were no longer statistically significant. However, in the crude analyses, both the medium and the high tertile had significantly less percentage motile spermatozoa compared with the low *p*,*p*′-DDE tertile. This was corroborated by the adjusted analyses with a significant difference between the high and the low tertiles and a tendency toward less spermatozoa in the medium tertile compared with the reference. None of the differences between groups were, however, corroborated by statistically significant trends on continuous exposures (Table 5).

There were no indications of threshold effects or dose–response relationships between *in utero* exposure to ΣPCB, ΣDL-PCB, and *p*,*p*′-DDE and semen quality measures or reproductive hormone levels at the examined exposure ranges.

CASA results supported the results from the conventional semen analysis assessment (Supplementary Tables 1, 2 and 3, see section on supplementary data given at the end of this article). In addition, complete case analyses (Supplementary Tables 4, 5 and 6) and sensitivity analyses with different imputation models (fewer predictors, more predictors, and a model where the best transformation of outcomes and variables were used in stead of ln-transformations in the multiple regression model) all supported the results from the main imputation model, except that the ‘best transformation’ model indicated a positive association between continuous *p*,*p*′-DDE exposure and sperm concentration (Supplementary Table 7), which was even more pronounced in the complete case analyses (Supplementary Table 6). In addition, complete case analyses also suggested that higher *in utero* exposure to *p*,*p*′-DDE was statistically, significantly associated with higher total sperm count in adult life (Supplementary Table 6).

When comparing PCB and *p*,*p*′-DDE levels, according to level of participation in the study, there were no statistically significant differences in exposure levels between the sons who participated in the physical examination, those who only filled in questionnaires, and those who were lost to follow-up (*P*=0.11, *P*=0.12, and *P*=0.48 for ΣPCB, ΣDL-PCB, and *p*,*p*′-DDE, respectively; Table 6). Furthermore, there were no differences in maternal pre-pregnancy BMI, parity, social class, serum total lipid level, alcohol consumption during pregnancy or sons' BMI, or smoking status. There was a tendency toward a higher degree of young men who had a history of reproductive tract disease among those who participated in the physical examination compared with those who only filled in the questionnaire (*P*=0.06). The mothers of sons who did not participate in follow-up were statistically significantly younger than mothers of sons who participated in the physical examination. In addition, we identified less reported smoking during pregnancy among the mothers whose sons participated in the physical examination compared with those who did not participate in follow-up (Table 6).

## Discussion

In this study, we found that semen quality measures and reproductive hormone levels were not consistently, adversely related to *in utero* exposure to either ΣPCBs, ΣDL-PCBs, or *p*,*p*′-DDE when using the approach of analysing one chemical/one chemical group at a time.

A study on mice exposed *in utero* and postnatally through lactation to a mixture of PCB-101 and PCB-118 to doses estimated to represent 0, 1, 10, and 100 μg PCB/kg per day suggested that perinatal PCB exposure was associated with decreased testis weight and sperm viability but not with sperm concentration. At the highest PCB dose, a significant reduction in anogenital distance (AGD) was also observed compared with controls (Pocar *et al*. 2012). Another study, in which rat dams were treated with a single dose of 375 μg PCB-118/kg on gestational day 6 found that *in utero* exposure to PCB-118 was associated with increased AGD and reduced testis and epididymis weights in the male offspring. In addition, sperm concentration, daily sperm production, and sperm counts were significantly reduced compared with unexposed controls. In this study, exposure levels were ∼100-fold higher than human exposure levels in breast milk (Kuriyama & Chahoud 2004).

We did not find any inverse associations between maternal pregnancy week 30 serum levels of PCB or *p*,*p*′-DDE and testis size or sperm count. This supports previous epidemiological studies on associations between *in utero* exposure and male reproductive organ development, which generally found weak or no effects. Two nested case–control studies found that maternal pregnancy levels of *p*,*p*′-DDE were not statistically significantly associated with increased risk of hypospadias and cryptorchidism (Longnecker *et al*. 2002, Bhatia *et al*. 2005), and two nested case-control studies on placental- and maternal serum concentrations of PCB during pregnancy found no increased risk of cryptorchidism (McGlynn *et al*. 2009, Virtanen *et al*. 2012). However, indications of increased risks of hypospadias and cryptorchidism in relation with *in utero* exposure to PCB and *p*,*p*′-DDE have also been reported (Brucker-Davis *et al*. 2008, McGlynn *et al*. 2009, Rignell-Hydbom *et al*. 2012). We cannot exclude that the lack of statistically significant associations in the current study might be due to a lack of exposure contrast in the data. However, median maternal serum POP levels in our study were comparable with those of Rignell-Hydbom *et al*. (2012), who found a tendency toward an association with *p*,*p*′-DDE levels. The data collection period in the Rignell–Hydbom study spans from 1986 until 2002, while maternal POP levels during pregnancy in the studies by Longnecker *et al*. (2002), Bhatia *et al*. (2005), and McGlynn *et al*. (2009) were substantially higher than the present concentrations, corresponding to a heavier POP load during the 1960s when specimens for these studies were collected.

The study by Guo *et al*. (2000) on young males prenatally exposed to high levels of PCBs and polychlorinated dibenzofurans (PCDFs) as a result of the Yu Cheng oil-disease suggested long-term adverse effects on sperm quality in terms of abnormal morphology, decreased sperm motility, and reduced oocyte penetration capacity (Guo *et al*. 2000).

We did not find any consistent associations between *in utero* exposure to PCBs or *p*,*p*′-DDE and sperm morphology or sperm motility in the present study, except from the statistically significant less percentage motile spermatozoa in the high *p*,*p*′-DDE tertile compared with the reference tertile, which was not corroborated by a significant trend on the continuous exposure. The males born to exposed Yu Cheng women were exposed to high levels of PCBs and PCDFs during fetal development because of their mothers' accidental ingestion of cooking oil contaminated with these POPs. As the young men in our cohort were only exposed to background POP levels in the late 1980s, differences between the findings of the Guo *et al*. (2000) study and our study are likely to be explained by differences in exposures levels.

Another study investigated the possible male reproductive health consequences of perinatal exposure to dioxins in a cohort of men born to mothers who were exposed to dioxins as a result of the trichlorophenol plant explosion near Seveso, Italy in 1976. This study found that sons who were exposed both *in utero* and through breast feeding had significantly lower sperm concentration, total sperm count, progressive motility, and total motile count than the unexposed controls. There were no differences in semen quality measures between sons, who were only exposed *in utero*, and controls (Mocarelli *et al*. 2011). In our study, we observed no associations between the dioxin-like PCBs (PCB-118 and PCB-156) and any of the investigated male reproductive health outcomes. This could be due to differences in exposure levels between the two studies or to a higher contribution by postnatal exposure, which was not assessed in the present study.

During the last decade, more focus has been turned toward “the mixture effect” – that compounds with potential hormone-modulating effects may only contribute with minor effects at the individual compound level, and that a health effect may only appear in concert with other chemicals. Animal studies have suggested that exposure to a mixture of different compounds can produce effects on male reproductive health at concentrations where there are no associations between the individual compounds and the male reproductive health outcome (Christiansen *et al*. 2008, Kortenkamp 2008), and studies have indicated that the mixture of compounds rather than individual chemical exposures may contribute to effects on male reproductive health (Krysiak-Baltyn *et al*. 2012). With the mixture effect in mind, we cannot exclude that PCB and *p*,*p*′-DDE exposure in concert with other potential hormone-modulating compounds may result in adverse associations with male reproductive health, although overall no statistically consistent significant results were found in the current study using the single chemical/chemical group approach.

Complete case results and the multiple regression analyses, in which we checked ln-transformed results with a model using the most optimal transformations of the outcome variables, suggested a positive association between maternal serum levels of *p*,*p*′-DDE during pregnancy and the sperm concentration of sons in adult life. Similar findings have been reported independently in subsets of the study populations of two studies assessing the effects of adult male serum levels of *p*,*p*′-DDE on male reproductive health (Toft *et al*. 2006, Haugen *et al*. 2011). Due to the antiandrogenic properties of *p*,*p*′-DDE, this was opposite of what we expected and may be a result of multiple testing.

A previous study on this cohort of young men found inverse associations between maternal pregnancy week 30 serum levels of perfluorooctanoic acid (PFOA) and sperm concentration and total sperm count and positive associations to LH and FSH (Vested *et al*. 2013). A sub-analysis adjusting for continuous PFOA did not alter the directions or significance levels of the results for either ΣPCB, ΣDL-PCB, or *p*,*p*′-DDE analyses (data not shown).

This study is to our knowledge the first population-based follow-up study to report on associations between *in utero* exposure to PCBs and *p*,*p*′-DDE and semen quality and reproductive hormone levels in early adult life.

The longitudinal design of the present study has a major strength compared to cross-sectional studies attempting to study the same hypothesis. It allowed us to estimate the effects of exposure to POPs during a proposed critical window of exposure to exogenous compounds with semen quality measures and reproductive hormone levels later in life.

A possible limitation of this study includes the risk of selection bias, which could be present if study participation was associated with both POP exposure and reproductive outcomes. It has been shown by former studies that men experiencing fertility problems are more willing to participate in studies investigating male reproductive health outcomes (Bonde *et al*. 1996). The participants in this cohort were young and for the most part had no reproductive experience. Thus, it is unlikely that participation in this study was related to fecundity. However, there was a tendency toward more participants having a history of reproductive tract disease among the participants who attended the physical examination compared with the young men who only filled in the internet based questionnaire (*P*=0.06). However, there is no reason to believe that a potential oversampling of young men with a history of reproductive tract disease would be related to exposure and thus unlikely that this would have biased the results. Participation rate of the physical examination was 38%. However, losses to follow-up were not associated with ΣPCB, ΣDL-PCB, or *p*,*p*′-DDE exposure levels as there were no differences in exposure levels between the sons who participated in the physical examination, those who only filled in internet-based questionnaires, and those who did not participate in the follow-up study (Table 6). In addition, sons were unaware of their mothers' serum POP levels during pregnancy. Hence, we do not believe that selection bias is a major concern.

Because of the long half-lives of POPs in the human body, one blood sample measurement in pregnancy week 30 is considered a satisfactory proxy for fetal exposure during pregnancy.

A median lipid-adjusted *p*,*p*′-DDE level of 288 ng/g lipid as measured in serum samples from pregnancy week 30 in 1988–1989 in the current study is comparable with *p*,*p*′-DDE levels measured in male partners of infertility couples in USA in 2000–2001 (275 ng/g lipid) (Hauser *et al*. 2003), and considerably higher compared with Scandinavian levels measured in Northern and Southern Norway in 2001 (66–81 ng/g lipid; Haugen *et al*. 2011). The median PCB-153 lipid-adjusted concentration was 165 ng/g lipid in the current study, which is somewhat higher than American and Scandinavian background levels in the 2000s (44–68 ng/g lipid; Hauser *et al*. 2003, Richthoff *et al*. 2003, Haugen *et al*. 2011). Thus, our findings are potentially relevant to contemporary generations.

Despite indications from previous studies of associations between POP levels in adult life and male reproductive health, the present results do not suggest long-term effects of *in utero* exposure to PCBs or *p*,*p*′-DDE alone on semen quality measures or reproductive hormone levels; however it cannot be excluded that exposure to PCBs and *p*,*p*′-DDE in combination with other endocrine-modulating compounds may have adverse effects on the male reproductive system.

## Supplementary data

This is linked to the online version of the paper at http://dx.doi.org/10.1530/REP-13-0488.

## Figures and Tables

**Table 1:** Maternal serum levels of six PCB congeners and *p*,*p*′-DDE.

	**Median** (range)
Wet weight serum concentrations (ng/ml)	
PCB-118	0.17 (0.05–0.59)
PCB-138	0.75 (0.14–2.58)
PCB-153	1.46 (0.27–5.81)
PCB-156	0.10 (0.02–0.34)
PCB-170	0.38 (0.08–1.14)
PCB-180	0.71 (0.15–2.44)
*p*,*p*′-DDE	2.55 (0.23–17.59)
Lipid-adjusted serum concentrations (ng/g lipid)	
PCB-118	20 (6–60)
PCB-138	88 (16–263)
PCB-153	165 (31–591)
PCB-156	12 (2–37)
PCB-179	43 (9–148)
PCB-180	81 (18–277)
*p*,*p*′-DDE	288 (31–2208)

**Table 2:** Characteristics of 166 young men by tertiles of maternal pregnancy week 30 serum ΣPCB and *p*,*p*′-DDE concentrations.

	**ΣPCB**				***p*,*p*′-DDE**			
	Low (2.14–8.02pmol/ml), *n*=55	Medium (>8.02–11.47pmol/ml), *n*=56	High (>11.47–34.99pmol/ml), *n*=55	*P* value	Low (0.73–6.30pmol/ml), *n*=55	Medium (>6.30–10.63pmol/ml), *n*=56	High (>10.63–55.32pmol/ml), *n*=55	*P* value
Characteristics of sons								
BMI (kg/m^2^), median (p25–p75)	22.7 (20.9–24.4)	22.6 (20.5–24.5)	23.0 (20.6–24.1)	0.77^a^	22.3 (20.3–24.0)	22.8 (20.8–24.4)	22.8 (20.9–24.2)	0.64^a^
History of reproductive tract disease^b^	7 (13.7)	8 (16.0)	7 (13.2)	0.91^c^	9 (17.7)	7 (13.7)	6 (11.5)	0.67^c^
Current/ occasional smoker	26 (49.1)	25 (49.0)	29 (54.7)	0.80^c^	27 (51.9)	29 (54.7)	24 (46.2)	0.67^c^
Characteristics of mothers								
Mother smoking during pregnancy	18 (33.3)	15 (27.3)	18 (35.3)	0.65^c^	16 (30.2)	18 (33.3)	17 (32.1)	0.94^c^
Socioeconomic status (Total annual household income 1987)								
<200 000 DKK	23 (46.9)	21 (39.6)	15 (30.0)	0.22^c^	21 (43.8)	21 (38.9)	17 (34.0)	0.61^c^
>200 000 DKK	26 (53.1)	32 (60.4)	35 (70.0)	0.22^c^	27 (56.3)	33 (61.1)	33 (66.0)	0.61^c^
Serum total lipid level (g/l), median p25–p75)	8.2 (7.5–9.0)	8.6 (7.6–9.3)	9.0 (7.8–10.3)	0.01^a^	8.3 (7.5–9.3)	8.6 (7.6–9.6)	8.5 (7.7–10.1)	0.43^a^
Characteristics of sons' semen and serum								
Duration of abstinence (h)								
≤48	23 (41.8)	33 (58.9)	35 (64.8)	0.04^c^	23 (41.8)	35 (62.5)	33 (61.1)	0.05^c^
≥49	32 (58.2)	23 (41.1)	19 (35.2)	0.04^c^	32 (58.2)	21 (37.5)	21 (38.9)	0.05^c^
Min. from ejaculation to semen analysis, median (p25–p75)	43.5 (30.0–55.0)	40.0 (27.5–55.0)	50.0 (35.0–60.0)	0.25^a^	37.5 (25.0–50.0)	45.0 (30.0–57.5)	50.0 (35.0–55.0)	0.11^a^
Spillage at semen sampling	14 (27.5)	14 (26.4)	16 (30.8)	0.88^c^	10 (19.6)	15 (28.3)	19 (36.5)	0.16^c^
Time for blood sampling								
0730–0929	19 (35.2)	15 (27.3)	19 (35.2)	0.60^c^	18 (32.7)	16 (29.1)	19 (35.9)	0.79^c^
0930–1129	29 (53.7)	29 (52.7)	24 (44.4)	0.60^c^	31 (56.4)	28 (50.9)	23 (43.4)	0.79^c^
1130–	6 (11.1)	11 (20.0)	11 (20.4)	0.60^c^	6 (10.9)	11 (20.0)	11 (20.8)	0.79^c^

Numbers are based on complete case data and indicate number (percentage) unless otherwise stated.

a Differences across tertiles of ΣPCB and *p*,*p*′-DDE exposure where tested by Kruskal–Wallis test.

b History of reproductive tract disease include: inguinal hernia, varicocele, testicular hydrocele, incarcerated hernia, phimosis, torsio testis, clamydia, gonorrhea, and epididymitis combined into one variable (yes/no).

c Differences across tertiles of ΣPCB and *p*,*p*′-DDE exposure where tested by *χ* ^2^-test.

**Table 3:** Characteristics of semen, testicular size, and reproductive hormone for 173 young Danish men stratified by tertiles of maternal pregnancy week 30 serum ΣPCB concentrations (pmol/ml).

		**Median** (p25–p75)					**Adjusted percentage difference from low ΣPCB group** ^a^ (95% CI)			
**Parameter**	***n***	Low ΣPCB (*n*=58)	Medium ΣPCB (*n*=58)	High ΣPCB (*n*=57)	β-coeff (95% CI)^b^	Trend *P* value^b^	Medium ΣPCB	High ΣPCB	Adjusted ln β-coeff (95% CI)^a^ ^b^	Adjusted trend^a^ ^b^ *P* value
Sperm concentration (10^6^/ml)	173	37 (13–64)	37 (15–60)	42 (23–91)	0.22 (−1.50; 1.95)	0.80	−8 (−52; 36)	22 (−24; 68)	0.03 (−0.02; 0.07)	0.25
Total sperm count (10^6^)	129	123 (53–223)	130 (42–215)	92 (55–221)	−0.52 (−5.75; 4.70)	0.84	−12 (−65; 41)	18 (−38; 73)	0.02 (−0.03; 0.08)	0.44
Semen volume (ml)	129	3.0 (2.2–4.2)	3.1 (2.2–3.9)	2.8 (1.8–4.0)	−0.02 (−0.08; 0.05)	0.57	5 (−15; 26)	0 (−22; 21)	−0.002 (−0.02; 0.02)	0.86
Percentage progressive spermatozoa	173	66 (52–74)	64 (52–73)	65 (52–69)	−0.28 (−0.69; 0.12)	0.16	−1 (−10; 9)	−1 (−11; 9)	−0.003 (−0.01; 0.006)	0.49
Percentage motile spermatozoa	173	74 (66–80)	72 (66–76)	73 (66–76)	−0.21 (−0.51; 0.09)	0.17	−2 (−8; 5)	−2 (−8; 4)	−0.002 (−0.008; 0.004)	0.52
Percentage morphologically normal spermatozoa	161	8 (4–12)	9 (4–14)	9 (4–12)	0.05 (−0.13; 0.23)	0.58	4 (−43; 51)	−15 (−64; 34)	−0.01 (−0.06; 0.03)	0.59
Mean testicular volume (ml)	173	15 (11–20)	15 (13–20)	15 (11–20)	0.05 (−0.12; 0.22)	0.57	2 (−11; 16)	−2 (−16; 12)	0.003 (−0.01; 0.02)	0.71
Testosterone (nmol/l)	173	23 (19–26)	20 (17–23)	21 (18–27)	0.01 (−0.15; 0.18)	0.88	−11 (−21; −1)	−1 (−11; 10)	0.0004 (−0.01; 0.01)	0.94
Free testosterone (nmol/l)	173	0.55 (0.45–0.62)	0.47 (0.41–0.57)	0.53 (0.45–0.62)	−0.001 (−0.005; 0.002)	0.41	−11 (−22; −1)	−1 (−13; 10)	−0.0009 (−0.01; 0.01)	0.86
Estradiol (nmol/l)	173	0.10 (0.09–0.13)	0.09 (0.08–0.11)	0.10 (0.08–0.11)	−0.0006 (−0.002; 0.0003)	0.18	−16 (−28; −5)	−4 (−16; 8)	−0.003 (−0.01; 0.009)	0.64
LH (IU/l)	173	4.5 (3.3–6.0)	4.1 (3.1–4.9)	4.4 (3.6–5.7)	0.01 (−0.04, 0.07)	0.70	−13 (−28, 1)	−2 (−17; 13)	−0.0009 (−0.02; 0.01)	0.90
FSH (IU/l)	173	2.9 (2.0–4.1)	3.1 (2.3–4.6)	3.0 (2.4–4.3)	0.01 (−0.05; 0.08)	0.73	7 (−13; 27)	−1 (−20; 21)	0.001 (−0.02; 0.02)	0.90
Inhibin B (pg/ml)	173	219 (158–259)	225 (172–283)	221 (176–278)	0.95 (−1.65; 3.56)	0.47	5 (−11; 21)	8 (−8; 24)	0.008 (−0.007; 0.02)	0.28
SHBG (nmol/l)	173	26 (21–33)	27 (22–32)	27 (21–34)	0.19 (−0.12;0.49)	0.23	−4 (−17; 9)	−1 (−14; 13)	0.001 (−0.01; 0.01)	0.88

Note: p, percentile.

a Adjustment: all multiple regression results were adjusted for the history of reproductive tract disease, the son's BMI; son's smoking status, maternal smoking during pregnancy, socioeconomic status, and total blood lipid concentration. Sperm concentration, total sperm count, progressive spermatozoa, motile spermatozoa, semen volume, and testicular volume were adjusted for abstinence time, sperm concentration was adjusted for spillage during semen sample collection, progressive spermatozoa and motile spermatozoa were adjusted for time from ejaculation to semen analysis, and the reproductive hormones were adjusted for time of day of blood sampling.

b Trends on ΣPCB concentrations (continuous data) were tested by robust linear regression analysis (untransformed crude data) and multiple regression analysis (adjusted data).

**Table 4:** Characteristics of semen, testicular size, and reproductive hormone for 173 young Danish men stratified by tertiles of maternal pregnancy week 30 serum ΣDL-PCB concentrations.

		**Median** (p25–p75)					**Adjusted percentage difference from low ΣDL-PCB group** ^a^ (95% CI)			
**Parameter**	***n***	Low ΣDL-PCB (*n*=58)	Medium ΣDL-PCB (*n*=58)	High ΣDL-PCB (*n*=57)	β-coeff (95% CI)^a^	Trend *P* value^a^	Medium ΣDL-PCB	High ΣDL-PCB	Adjusted β-coeff (95% CI)^a^ ^b^	Adjusted trend *P* value^a^ ^b^
Sperm concentration (10^6^/ml)	173	37 (13–62)	36 (16–61)	42 (22–94)	4.03 (−15.23; 23.28)	0.68	−2 (−46; 43)	22 (−25; 70)	0.25 (−0.24; 0.73)	0.32
Total sperm count (10^6^)	129	123 (27–215)	93 (45–198)	128 (54–265)	11.91 (−41.37; 65.18)	0.66	2 (−52; 56)	33 (−23; 90)	0.26 (−0.36; 0.87)	0.41
Semen volume (ml)	129	3.0 (2.2–4.2)	3.1 (2.1–3.9)	3.0 (2.0–4.1)	−0.004 (−0.68; 0.67)	0.99	7 (−14; 28)	9 (−13; 31)	0.07 (−0.17; 0.30)	0.57
Percentage progressive spermatozoa	173	66 (52–74)	65 (52–74)	65 (52–69)	−2.25 (−6.80; 2.29)	0.33	2 (−8; 12)	−1 (−11; 9)	−0.02 (−0.13; 0.09)	0.71
Percentage motile spermatozoa	173	74 (63–79)	74 (69–80)	72 (64–76)	−1.99 (−5.58; 1.60)	0.28	3 (−3; 9)	−2 (−9; 4)	−0.02 (−0.09; 0.05)	0.56
Percentage morphologically normal spermatozoa	161	9 (5–13)	7 (3–12)	9 (6–13)	0.24 (−1.88; 2.36)	0.82	−21 (−68; 25)	−15 (−65; 35)	−0.16 (−0.70; 0.39)	0.56
Mean testicular volume (ml)	173	15 (11–20)	18 (13–20)	15 (12–20)	0.75 (−1.07; 2.57)	0.42	9 (−4; 23)	4 (−10; 18)	0.05 (−0.09; 0.20)	0.49
Testosterone (nmol/l)	173	22 (18–25)	21 (17–23)	21 (19–27)	−0.01 (−1.94; 1.92)	0.99	−7 (−18; 3)	−2 (−13; 9)	−0.003 (−0.12; 0.11)	0.95
Free testosterone	173	0.53 (0.43–0.61)	0.51 (0.43–0.60)	0.54 (0.43–0.61)	−0.01 (−0.06; 0.03)	0.60	−4 (−16; 7)	−2 (−13; 10)	−0.01 (−0.13; 0.11)	0.85
Estradiol (nmol/l)	173	0.10 (0.08–0.12)	0.09 (0.08–0.11)	0.10 (0.08–0.11)	−0.006 (−0.02; 0.004)	0.25	−6 (−18; 6)	−4 (−17; 8)	−0.04 (−0.17; 0.09)	0.50
LH (IU/l)	173	4.5 (3.6–5.8)	4.1 (3.1–5.1)	4.4 (3.6–5.6)	0.09 (−0.57; 0.76)	0.78	−11 (−26; 4)	−2 (−18; 13)	−0.005 (−0.17; 0.16	0.95
FSH (IU/l)	173	2.9 (2.0–4.3)	3.1 (2.3–4.5)	3-0 (2.4–4.3)	0.001 (−0.73; 0.73)	1.00	8 (−12; 29)	1 (−20; 22)	−0.006 (−0.23; 0.21)	0.95
Inhibin B (pg/ml)	173	219 (158–261)	225 (181–281)	224 (159–277)	13.74 (−16.97; 44.45)	0.38	4 (−12; 20)	7 (−10; 23)	0.11 (−0.06; 0.29)	0.20
SHBG (nmol/l)	173	26 (22–34)	26 (21–31)	28 (22–35)	1.47 (−2.20; 5.15)	0.43	−9 (−23; 4)	−3 (−17; 10)	−0.01 (−0.15; 0.13)	0.89

Note: p, percentile.

a Adjustment: all multivariable regression results were adjusted for the history of reproductive tract disease, the son's BMI; son's smoking status, maternal smoking during pregnancy, socioeconomic status, and total lipid. Sperm concentration, total sperm count, progressive spermatozoa, motile spermatozoa, semen volume, and testicular volume were adjusted for abstinence time; sperm concentration was also adjusted for spillage during semen sample collection; progressive spermatozoa and motile spermatozoa were also adjusted for time from ejaculation to semen analysis; reproductive hormones were also adjusted for time of day of blood sampling.

b Trends on Σdioxin-like PCB concentrations (continuous data) were tested by robust linear regression analysis (untransformed crude data) and multiple regression analysis (adjusted data).

**Table 5:** Characteristics of semen-, testicular size-, and reproductive hormone for 173 young Danish men stratified by tertiles of maternal pregnancy week 30 serum *p*,*p*′-DDE concentrations.

		**Median** (p25–p75)					**Adjusted** ^a^ **percentage difference from low *p*,*p*′-DDE group** (95% CI)			
**Parameter**	***n***	Low *p*,*p*′-DDE (*n*=58)	Medium *p*,*p*′-DDE (*n*=58)	High *p*,*p*′-DDE (*n*=57)	β-coeff (95% CI)^b^	Trend *P* value^b^	Medium *p*,*p*′-DDE	High *p*,*p*′-DDE	Adjusted β-coeff (95% CI)	Adjusted trend *P* value^a^ ^b^
Sperm concentration (10^6^/ml)	173	40 (16–62)	33 (18–63)	40 (16–93)	0.83 (−0.17; 1.83)	0.10	−5 (−51; 40)	14 (−32; 61)	0.01 (−0.006; 0.03)	0.15
Total sperm count (10^6^)	129	122 (60–222)	113 (43–199)	117 (49–267)	0.80 (−1.73; 3.34)	0.53	−14 (−68; 40)	9 (−48; 66)	0.01 (−0.01; 0.03)	0.38
Semen volume (ml)	129	3.2 (2.4–4.3)	2.7 (1.9–3.5)	3.0 (2.0–4.1)	−0.01 (−0.03; 0.01)	0.41	−13 (−33; 8)	−8 (−30; 14)	−0.002 (−0.01; 0.007)	0.71
Percentage progressive spermatozoa	173	70 (59–74)	63 (49–70)	65 (53–70)	−0.04 (−0.24; 0.15)	0.66	−7 (−17; 3)	−5 (−15; 5)	0.0005 (−0.004; 0.005)	0.81
Percentage motile spermatozoa	173	76 (71–81)	72 (66–77)	72 (63–76)	−0.13 (−0.29; 0.03)	0.11	−5 (−11; 1)	−7 (−13; −1)	−0.001 (−0.004; 0.001)	0.34
Percentage morphologically normal spermatozoa	173	8 (4–14)	7 (4–13)	10 (6–12)	0.02 (−0.09; 0.12)	0.74	−6 (−59; 46)	11 (−45; 68)	−0.002 (−0.03; 0.03)	0.90
Mean testicular volume (ml)	173	15 (11–20)	15 (12–20)	15 (13–20)	0.03 (−0.04; 0.11)	0.41	5 (−9; 19)	8 (−6; 22)	0.002 (−0.004; 0.008)	0.50
Testosterone (nmol/l)	173	21 (18–24)	21 (17–24)	21 (19–27)	0.07 (−0.02; 0.16)	0.13	−2 (−12; 9)	7 (−3; 18)	0.003 (−0.001; 0.008)	0.13
Free testosterone	173	0.51 (0.44–0.60)	0.54 (0.42–0.61)	0.52 (0.44–0.61)	0.001 (−0.0007; 0.003)	0.24	2 (−9; 13)	3 (−8; 14)	0.003 (−0.002; 0.007)	0.29
Estradiol (nmol/l)	173	0.10 (0.08–0.11)	0.09 (0.08–0.12)	0.10 (0.08–0.12)	0.00009 (−0.0003; 0.0005)	0.67	−4 (−16; 8)	−1 (−14; 11)	0.0009 (−0.004; 0.006)	0.73
LH (IU/l)	173	4.3 (3.5–5.4)	4.4 (3.7–5.5)	4.4 (3.1–5.8)	0.02 (−0.008; 0.05)	0.17	−5 (−20; 11)	−5 (−20; 11)	0.002 (−0.005; 0.009)	0.57
FSH (IU/l)	173	2.9 (2.2–4.1)	3.0 (2.1–4.4)	3.1 (2.1–4.5)	0.0001 (−0.03; 0.03)	1.00	−1 (−21; 19)	−3 (−24; 17)	−0.0002 (−0.009; 0.009)	0.97
Inhibin B (pg/ml)	173	226 (158–271)	209 (168–255)	231 (180–288)	−0.07 (1.26; 1.12)	0.91	−5 (−20; 11)	8 (−8; 24)	0.001 (−0.006; 0.008)	0.75
SHBG (nmol/l)	173	25 (20–34)	24 (20–30)	30 (24–37)	0.09 (−0.09; 0.26)	0.33	−8 (−21; 5)	12 (−2; 25)	0.003 (−0.003; 0.009)	0.37

Note: p, percentile.

a Adjustment: all multivariable regression results were adjusted for the history of reproductive tract disease, the son's BMI; son's smoking status, maternal smoking during pregnancy, and socioeconomic status. Sperm concentration, total sperm count, progressive spermatozoa, motile spermatozoa, semen volume, and testicular volume were adjusted for abstinence time; sperm concentration was also adjusted for spillage during semen sample collection; progressive spermatozoa and motile spermatazoa were also adjusted for time from ejaculation to semen analysis; reproductive hormones were also adjusted for time of day of blood sampling.

b Trends *p*,*p*′-DDE concentrations (continuous data) were tested by robust linear regression analysis (untransformed crude data) and multiple regression analysis (adjusted data).

**Table 6:** Characteristics of mothers and sons at baseline (pregnancy week 30) and follow-up according to the level of participation.

**Characteristics**	**Participating in physical examination, *n*=176**	**Filling in questionaire only, *n*=157**	**Not in follow-up, *n*=143**	***P* value**
Maternal				
ΣPCBs (pmol/ml), median (p25–p75)	10.1 (7.3–12.9)	10.4 (7.6–13.0)	9.0 (6.6–12.2)	0.12^a^
ΣDioxin-like-PCBs (pmol/ml), median (p25–p75)	0.8 (0.6–1.1)	0.8 (0.6–1.1)	0.8 (0.6–1.0)	0.11^a^
*p*,*p*′-DDE (pmol/ml), median (p25–p75)	8.2 (5.2–12.4)	7.3 (4.9–11.2)	8.0 (5.1–11.9)	0.48^a^
Maternal age (years), mean (s.d.)	29.6 (4.4)	29.0 (4.2)	28.3 (4.5)	0.03^b^
Prepregnancy BMI (kg/m^2^), median (p25–p75)	21.0 (19.8–22.3)	20.7 (19.5–22.7)	21.2 (19.1–23.1)	0.77^a^
Nulliparous, *n* (%)	112 (64)	96 (61)	83 (58)	0.60^c^
Smoking during pregnancy, *n* (%)	55 (33)	53 (37)	63 (47)	0.03^c^
High social class, *n* (%)	96 (60)	87 (64)	77 (61)	0.78^c^
Blood total lipid level (g/l), median (p25–p75)	8.6 (7.7–9.6)	8.8 (7.9–10.0)	8.7 (7.9–9.6)	0.33^a^
Alcohol during pregnancy (g/day), median (p25–p75)	2.0 (0.4–4.6)	1.5 (0–4.4)	1.5 (0–3.5)	0.53^a^
Male offspring follow-up				
BMI (kg/m^2^), median (p25–p75)^d^	22.2 (20.5–24.1)	22.4 (20.7–24.4)	–	0.50^e^
Current/occasional smoker, *n* (%)	83 (50)	66 (43)	–	0.24^c^
History of reproductive tract disease, *n* (%)	23 (14)	11 (7)	–	0.06^c^

a Kruskal–Wallis test.

b One-way ANOVA test.

c *χ* ^2^-test.

d BMI is calculated from anthropometric measures based on self-reported values.

e Wilcoxon rank-sum test.
